# Real models and virtual simulators in otolaryngology: review of literature

**DOI:** 10.1590/S1808-86942010000100021

**Published:** 2015-10-17

**Authors:** João Flávio Nogueira Júnior, Daniel Nogueira Cruz

**Affiliations:** 1 Medical otorhinolaryngologist; 2 Medical resident in otorhinolaryngology, Sao Paulo Federal University - UNIFESP. Otorhinolaryngological and Ophthalmological Institute of Fortaleza (Instituto de Otorrinolaringologia e Oftalmologia de Fortaleza - IOF)

**Keywords:** teaching materials, models, computer simulation, anatomic

## Abstract

**Introduction and Aims:**

Real models and virtual simulators have been used with positive results in several fields of medicine. These new devices can enhance teaching, learning and also training in Otolaryngology, reducing associated costs and potentially reducing medical errors. We reviewed the literature on the real and virtual models and simulators used for education and training in our medical specialty, discussing some of them and the results achieved with such instruments. Moreover, we also discuss the future perspectives in education and training in our medical specialty.

**Methods:**

Literature review.

**Conclusions:**

Otolaryngology, a clinical and surgical field of medicine, should be at the forefront of this technological revolution. In our specialty, real models and virtual simulators and environments have a great teaching and learning potential. With equipment costs dropping, thanks to technological development, these tools tend to become increasingly more popular.

## INTRODUCTION

Real and virtual simulator models are in use in several areas of medicine, such as in anesthesiology, which for some time now has applied models for simulations and training of procedures such as orotracheal intubation, spinal anesthesia, and virtual simulators for training residents to carry out anesthetic procedures.^1^,^2^

Simulator models for basic procedures in other specialties have also been developed and used more recently for teaching and ongoing learning of physicians.^1^,^2^

This process gained force from 1999 onwards when the American Medical Institute published the paper: “To Err is Human: Building a Safer Health System.” This study presented alarming estimates about the North-American health system, particularly on medical error. For the first time a published paper showed that from 44 to 98 thousand patients died every year in the United States because of possible medical errors.^3^

After this article was published, the North-American medical community put in place several mechanisms aimed at reducing medical errors, improving healthcare and generating patient satisfaction with healthcare. As suggestions, the medical societies of that country recommended more intense training of medical students, medical residents, and practicing physicians with frequent recycling with simulations, similar to other professional fields such as civil aviation and the armed forces, where simulation is essential for training and for safety procedures.^2^, ^3^, ^4^

Published studies have shown that physicians trained with clinical or surgical simulators may provide objective performance data, monitoring their progress on the learning curve. Training experience may also be extended and measured in absolute terms, diversity and case complexity, as both real life models and virtual simulators can be developed for practically every condition and disease; a physician may repeat a procedure several times in a virtual software without any risk for the healthcare professional or the virtual patient.^2,^^5,^^6,^^7,^^8^

Real surgical or clinical simulation models are being developed - particularly in developed countries - based on anatomical images of real patients (computed tomography and magnetic resonance imaging). These models - based on real patients - may provide opportunities for anticipating the procedures that will be done in those patients, helping minimize eventual risks and complications.^9,^^10,^^11^

In the majority of Brazilian centers, training of medical residents in otorhinolaryngology includes theory classes, monitoring patients in an outpatient setting, video sessions of surgical procedures, dissection of recent cadavers, direct observation of surgical procedures, and carrying out procedures and surgery under supervision in training hospitals.^12^

Usually long training periods are required for training able professionals; unfortunately, this process has its restrictions in certain locations because of lack of patients, procedures and anatomical specimens for dissection in our country.^12^

Because of these issues - common to many countries - several authors believe that the future of medical training, especially in surgical specialties, will include real or virtual simulators for acquiring, maintaining and evaluating knowledge and abilities.^13^, ^14^, ^15^, ^16^, ^17^

There are already models and centers in Brazil that use simulators for teaching and training in otorhinolaryngology.

## OBJECTIVES

The objectives of our study were:

a) To review the current literature about the main models and real or virtual simulators used in teaching and training in otorhinolaryngology, and to discuss the results attained by using these tools.
b) To discuss the future perspectives in teaching and training in otorhinolaryngology.

## REVIEW OF THE LITERATURE

We will didactically divide our review according to the three main areas of knowledge in our specialty: otology, rhinology, and laryngology, presenting the main and most recent teaching and training real and virtual models and simulators.

### Otology

Otology has seen many developments in the use of simulators for teaching and training. There are many models and simulators ranging from simple real models for training in otoscopy to virtual simulators for temporal bone dissection.

**Figure 1:**
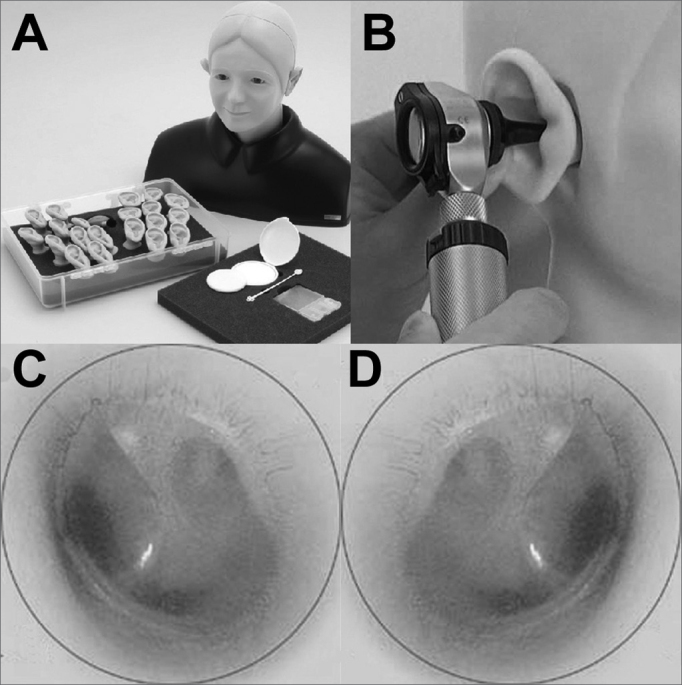
Otoscopy teaching and training model. A: model with head and several mobile parts for many disease types and otoscopies. B: practicing otoscopy in a model. C: example of a normal otoscopy of a left tympanic membrane in a model. D: example of a normal otoscopy of a right tympanic membrane in a model. Note the light triangle, the membrane with a slightly pearly sheen, and a view of part of the ossicular chain and auditory tube by transparency.

A recent Japanese model is used in some international centers for teaching otoscopy. It provides several possibilities, such as sinuous outer ear canals, tympanic membrane perforation, colesteatoma and tympanic glo-mus. This model is used in developed countries where few patients with ontological conditions are available.18 It has a fixed unit and a mobile head and unit consisting of an outer ear canal and a middle ear that can be exchanged and examined indefinitely (Fig. 1).

Although not expensive, it is still not available in Brazil. A recent study has shown that the results of motor training and the evaluation of disease in these models are comparable with the results attained when using real patients.^18^

There are also virtual simulators for temporal bone dissection. These simulators offer unlimited dissection with different types of virtual drills and tools, based on virtual reality concepts and direct interaction mechanisms with users, such as force feedback.^19^,^20^

Since the 1990s, computer software for temporal bone dissection has been developed and applied with relative success in medical residency program in many countries.^19^,^20^

The more recent virtual simulators have been developed in the United States at Ohio University, and also in Germany. A North-American simulator - the virtual temporal bone - is being validated, and is being used at several institutions, among them a Brazilian institution; it is called the virtual temporal bone project.

**Figure 2:**
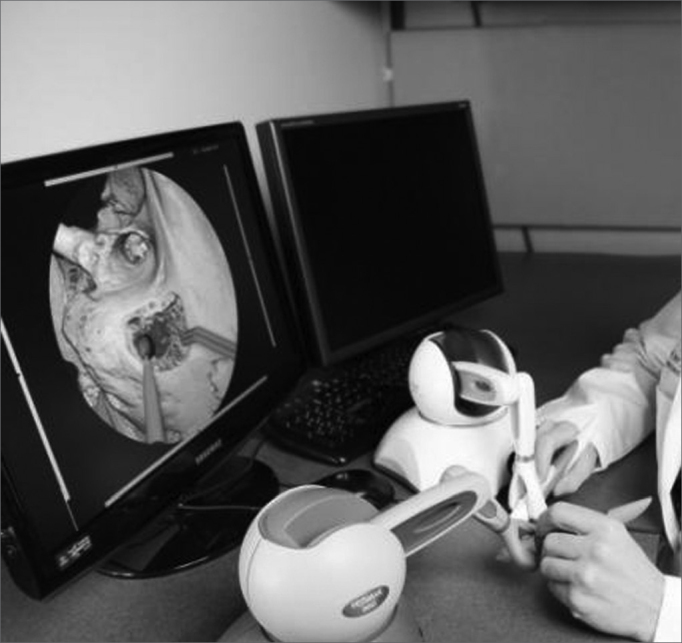
Virtual simulator of a temporal bone dissection. Virtual Temporal Bone Project. Note the controls that provide touch and force feedback.

It is a promising tool that provides structure recognition features and alarms when the trainee is close to anatomical landmarks such as the facial nerve, the dura-mater in the middle fossa, and the semicircular canals. It also features 3D graphic rendering to simulate various temporal bone textures and consistencies; an issue with this simulator is that it requires powerful graphic processing boards (at least 256 Mb non-shared memory) and force feedback controls (Fig. 2), both of which are still costly for Brazilian standards.^21^,^22^

**Figure 3:**
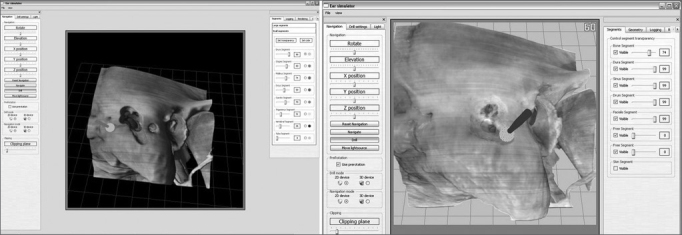
Computer screen image of virtual temporal bone dissection software. This German software may be downloaded free. Note well-defined textures and important anatomical structures, such as the facial nerve.

Another interesting simulator which was highlighted recently in the literature is being developed in Germany; it is distributed free of charge, and may be downloaded from the Internet.^22^ This software scores dissections from 0 to 10 according to whether important anatomical structures, such as the facial nerve, the dura-mater in the middle fossa, the ossicular bones, and the semicircular channels, are preserved (Fig. 3).

This German simulator requires high computer graphic processing power and dedicated controls, which may still limit its dissemination.^22^

Although several previous simulators have been validated, studies comparing these simulators with training in real temporal bone specimens have not yet been carried out with the aim of identifying eventual benefits -decreased complication rate - of simulator training vis-à-vis surgery in real patients. An interesting study was carried out in Canada in 2006 with the virtual temporal bone simulator; it showed that residents that had done virtual dissections reported improved learning of anatomy.^23^

The benefit of virtual simulators has been shown in other specialties, particularly for acquiring knowledge of anatomy; intensive training in virtual simulators helps decrease surgical time and complications.^2,^^4^, ^5^, ^6^, ^7^, ^8^

### Rhinology

Nasal and paranasal sinus functional endoscopic surgery is currently considered the gold standard for the treatment of several diseases in these areas. Handling the endoscope and surgical tools during these procedures is challenging due to the complex anatomy of this area and the proximity of important structures such as the brain, the eye, the carotid arteries, and the optic nerve, among others.^1^

**Figure 4:**
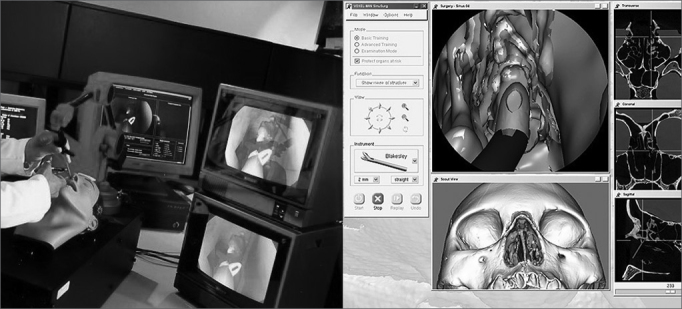
Virtual simulator of nasosinusal endoscopic surgery (ES3). Computer screen image of this software to the side. Note the possibility of changing virtually the tweezers, other tools, and dissection images.

The Lockheed Martin Corporation (Akron, Ohio, US) has recently developed a nasal and paranasal sinus endoscopic surgery virtual simulator - the ES3 - based on flight simulator concepts developed previously at this company for the North-American military forces.^1,^^10,^^24^, ^25^, ^26^ This simulator applied virtual reality and direct user interaction mechanisms, such as force feedback. Its use, however, remains restricted, even in the US, due to the high cost of equipment, which includes a powerful computer (Silicon Graphics Inc., Mountain View, Calif), force feedback controls (Phantom Omni), and a platform consisting of a replica of an endoscope, surgical tools, and a model plastic head (Fig. 4).

Several studies have described the development, validation and use of this simulator. Most of them show positive results of training of medical students and residents.^24^, ^25^, ^26^ This model, however, has certain disadvantages, such as high cost, the use of virtual images that are often confusing and have poor resolution, sensitive changes upon touching certain structures, and the impossibility of handing real tools and endoscopes used in traditional surgery.^26^,^27^

**Figure 5:**
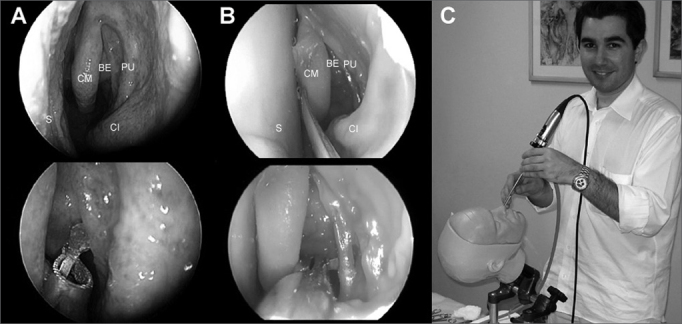
Images of the S.I.M.O.N.T. model. A: endoscopic image (0-degrees, 4mm) of an anatomical dissection of a recent cadaver. B: endoscopic image (0-degrees, 4mm) of an anatomical dissection in a S.I.M.O.N.T. model. Note the similarity between the structures and the use of real surgical tools also used in traditional surgery. (CM) middle turbinate; (BE) ethmoidal bulla; (PU) uncinate process; (CI) lower turbinate; (S) nasal septum. C: Physicians carrying out dissection of tasks required in the S.I.M.O.N.T. model. Note that the surgical instruments are the same as those in nasosinusal surgery.

Prodelphus (www.prodelphus.com; Recife, Pernam-buco, Brazil) and Brazilian physicians have developed a nasosinusal endoscopy dissection real model. This model is named S.I.M.O.N.T. (Sinus Model Otorhino-Neuro Trainer) and was generated based on pictures of real anatomical structures and recent cadaver dissection videos.^27^ In a recently published paper, ten otorhinolaryngologists of various levels of experience, dissected in this model after watching a video of a fresh cadaver dissection and reading the manual written for nasal and paranasal sinus endoscopic dissection. Results were promising, as 70% of participants reported gains in anatomical knowledge after training; this may be evidence of the efficacy of this model. The main reported advantages were: use of instruments similar to those used in real endoscopic surgery, and lack of inherent biological risk (Fig. 5). The main disadvantage was the possibility only of a single dissection in each nasal lateral wall.^27^,^28^ This model can also be applied to teaching minimally invasive techniques, such as balloon sinuplasty and endoscopic surgery of the cranial base.^28^ Furthermore, models may be customized according to specifications, such as placing specific diseases for training and the use of specific techniques for those conditions. Cost may also be a limiting factor for this model; depending on the type of model, the cost may range from 400.00 to 1,000.00 US dollars.

No studies have been undertaken about possibly improved surgical performance among physicians trained using this model. It is currently being used in nasosinusal endoscopic dissection courses for physicians in our country, and in other specialties = for instance ophthalmology - for training in endoscopic procedures such as dacriocistorrinostomias.^29^

**Figure 6:**
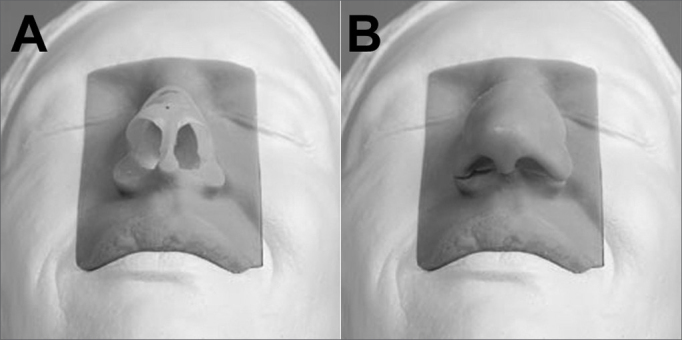
Rhinoplasty training model. A: model with exposed cartilage. B: model with synthetic skin covering cartilage. The white portion is a model of a fixed head. The skin-colored part is a module with synthetic skin and cartilage that may be dissected and touched.

There are also real models for training in rhinoplasty surgical techniques. Such a model has been developed in the UK for training in esthetic and functional techniques; a description has recently been published.^30^ The equipment was developed using synthetic material based on computed tomography images; it has a fully operating mobile unit and head including cartilage as found in the nose and artificial skin (Fig. 6). This model has not yet been validated, but it is currently being used in some centers to support dissection practices and surgical technique studies.^30^ We found no information about the cost of this model.

### Laryngology

Probably most of the models and simulators for teaching and training have been developed for otorhinolaryngology, especially those dealing with the airways.

The first models in laryngology were developed for training on cricostomy and tracheostomy techniques. There are several real (mannequin) models and virtual simulators, some of which have force feedback controls.^9,^^11,^^31^ Most of these models have been validated.

A recent paper on a training mannequin model was evaluated by 70 anesthesiologists in a randomized study on the time required for performing cricostomy and tracheostomy. Of these, 54 anesthesiologists that spent more training time on the models required less time to perform the procedure in real patients.^11^

An area of significant current interest is laryngeal surgery, especially phonosurgery; this area provides major challenges for physicians, who require intense training and to learn specific delicate handling techniques. A real model for laryngeal surgery training has been developed in the US; this model - the laryngeal dissection model - contains synthetic vocal folds placed as platforms to simulate the larynx.^31^ A recent paper validating this model described the development and training in surgery using the same instruments and microscope used in real surgery. Results were very forthcoming; physicians with little experience improved their performance after training on this model, and made fewer mistakes. There were fewer injuries of peripheral superficial tissues and surgical time was shorter compared to the group of residents that did no training on this specific model.^31^

There are also virtual programs such as the larynx virtual surgery software, which can simulate the vocal folds and glottic movements for training in phonosurgery.

## DISCUSSION

Although several factors are part of training, observing and performing procedures are an important component of medical training for competency and safety, especially in surgery.^3^, ^4^, ^5^ Unfortunately there have been few Brazilian studies on medical errors, although there is a general consensus among the North-American medical community that patient safety and satisfaction with surgical treatment needs to be improved. Such improvements are attained with intense medical training.^2,^^4,^^5,^^12^

There are no studies on the cost of training medical residents in Brazil. In the US, about 53 million dollars are spent in training general surgery residents. This number measures wasted surgical material, dissected anatomical specimens, increased surgical time due to learning, increased hospital stays of patients due to mistakes in procedures, more frequent surgical revisions, among others.^32^

As procedures become more complex, particularly with the use of endoscopes and minimally invasive techniques, training becomes more demanding, which raises costs. Real or virtual simulators may potentially control such costs.

Surgery is at the forefront in the development of computer simulators, not only for training, but also for recycling and ongoing training of surgeons; this is a proven approach.^5^ There are many benefits of this approach, since performance may be measured objectively in virtual simulators, and these may be reused many times, which avoids lack of availability or the paucity of procedures in some medical residency programs.

Virtual simulators may also mimic real patients about to undergo surgical procedures; in such cases, surgeons may test a procedure to identify possible difficulties and avoid complications.^5,^^14,^^19^

Otorhinolaryngology has also seen some advances in training simulation. Nasal and paranasal sinus endoscopic surgery is associated with perceptual and psychomotor practices to be acquired before undertaking real procedures. The inherent abilities for performing these techniques safely differ markedly from those of conventional surgery.^33^ Virtual simulators training has helped North-American medical residents in this development, without causing harm to or increasing the risk for patients.^1,^^14,^^15,^^18,^^19,^^33^

In Brazil we still do not have nasosinusal endoscopic surgery simulators available, mostly due to cost, since most teaching institutions do not have the financial means to fund these devices, which also involves constant maintenance. Nevertheless, we already have a nasosinusal endoscopic dissection real model available; some centers have the temporal bone dissection virtual software.

A few studies have shown that the ability to play musical instruments and electronic games is directly related with improved surgical performance, if approached correctly. Other authors have shown that practice in electronic games positively affects performance in videolaparoscopic surgery.^34^ Virtual simulators are similar to electronic games, and may thus potentially improve surgical performance.

We have seen that publications about virtual simulators in rhinology, otology and laryngology have evaluated their effectiveness in training; none of these studies, however, have been carried out in Brazil. A significant limitation commented in most of the papers is that the study samples are small, which may be due to the intrinsic limitations of these novel technologies. Although these sample are small, they have been genuinely accepted as a standard for studies on simulators in every form.^1^, ^14^, ^15^, ^18^, ^19^

The development of teaching and training models and simulators in otorhinolaryngology is limited only by imagination. Many directed applications may be created; the future of training and education in our specialty needs to be discussed to develop and apply these new tools. New procedures need to be developed and tested virtually; new safety tools and devices may also be assessed before being implemented, which will reduce costs and help develop our specialty.

New lines of research and trained professionals for writing virtual learning software will also gain importance; this may reduce distances, increase knowledge and save costs.

We recently participated in the first virtual conference in our specialty in Brazil. Within a virtual environment, professionals from different parts of the country gathered during a morning for learning and recycling of knowledge. Such initiatives, together with virtual learning and teaching software, have arrived to stay and should be incorporated by the teaching centers in our country.

## CONCLUSION

As a clinical and surgical specialty, otorhinolaryngology should place itself at the forefront of this technological revolution. Real models and virtual simulators, and virtual environments for teaching and learning, are potentially useful in our specialty. This approach tends to become popular as equipment costs fall and computer technology develops further.
